# Prevalence and Factors Associated with Multidrug-Resistant Tuberculosis (MDR-TB) among Presumptive MDR-TB Patients in Tigray Region, Northern Ethiopia

**DOI:** 10.1155/2019/2923549

**Published:** 2019-09-09

**Authors:** Kibriti Mehari, Tsehaye Asmelash, Haftamu Hailekiros, Tewolde Wubayehu, Hagos Godefay, Tadele Araya, Muthupandian Saravanan

**Affiliations:** ^1^Tigray Health Research Institute, Mekelle, Ethiopia; ^2^Department of Medical Microbiology and Immunology, Division of Biomedical Sciences, College of Health Sciences, Mekelle University, Mekelle, Ethiopia; ^3^College of Health Sciences, Aksum University, Axum, Ethiopia; ^4^Tigray Regional Health Bureau, Mekelle, Ethiopia

## Abstract

**Background:**

Tuberculosis (TB) is one of the major public health problems. There are alarming reports of increasing multidrug-resistant tuberculosis (MTR-TB) from various parts of the globe, including Ethiopia. This study was designed to determine the prevalence and factors associated with MDR-TB among presumptive MDR-TB cases in Tigray Regional State, Ethiopia.

**Methods:**

A cross-sectional study was conducted in Tigray Regional State from 2015 to 2016. Two hundred sputum samples were collected, transported, processed using 2% N-acetyl-L-cysteine-sodium hydroxide, and cultured in LJ medium. Besides, the microscopic examination was performed after ZN staining. Moreover, drug susceptibility test was done using molecular line probe assay. Descriptive statistics and binary and multivariable logistic regression were done. A statistical test was regarded as significant when the *P* value was <0.05.

**Results:**

The prevalence of MDR-TB was found to be 18.5%. About one-fourth (26.5%) of the study participants had sputum smear positive for acid-fast bacilli (AFB). TB culture was positive in 37% of the samples, and rifampicin mono-resistant cases accounted for 3.5% of the presumptive MDR-TB cases. Three (1.5%) were new MDR-TB cases, while the rest had been treated previously for TB. Most (63.5%) of the MDR-TB cases were from 15 to 44 years of age. Age was associated with MDR-TB with a crude odds ratio of 1.06 (CI: 1.02–1.10) and adjusted odds ratio of 1.06 (CI: 1.00–1.11).

**Conclusions:**

The prevalence of MDR-TB was found to be high. Preventive measures should be taken to prevent the transmission of MDR-TB in the community.

## 1. Introduction

Currently, TB is one of the major infectious diseases. About one-third of the world's population is infected with *M. tuberculosis*, and the majority of these infections occur in developing countries [1]. Anti-TB drugs such as rifampicin, isoniazid, ethambutol, streptomycin, and pyrazinamide have been used to treat the disease. Nonetheless, the occurrence of TB is reemerging, associated with human immunodeficiency virus, and resistance to the current anti-TB drugs is becoming a concern [2]. Notably, the emergence of drug-resistant TB is a threat to the world population, particularly resource-limited countries. MDR-TB is defined as resistance to at least isoniazid (INH) and rifampicin (RMP) [3].

Remarkably, in 2014, there were an estimated 480, 000 new cases of MDR-TB worldwide, and approximately 190,000 deaths from MDR-TB and more than half of these cases were in the developing world [4]. According to the 2014 World Health Organization (WHO) TB report, Ethiopia ranked 15th among 27 high-burden MDR-TB countries, with an estimated MDR rate of 1.6% (0.9%–2.8%) for new cases and 12 (5.6%–21%) for retreatment cases [5]. Furthermore, the studies conducted in some parts of Ethiopia have also revealed a high prevalence of MDR-TB (11.8% in the Oromia Region and 15.3% Amhara Region) [6, 7].

Several factors such as the burden of human immunodeficiency virus (HIV) infection, low socioeconomic status, and limited diagnostic and treatment facilities highly exacerbate the effect of MDR-TB in the developing world, including Ethiopia [8]. Furthermore, longer treatment regimen (about two years) and expensive and toxic drugs pose challenges in the programmatic management of MDR-TB in these countries [9]. On the other hand, TB and MDR-TB patients living in high-burden countries like Ethiopia stay in their communities for longer periods without being diagnosed or getting proper treatment. These altogether puts patients at risk for higher rates of treatment failure and deaths than the expected rates and also allows the easy spread of the disease to the public [10, 11]. Besides, as evidenced by few studies, behavioural and occupational factors like contact history with TB patient, contact with an MDR-TB patient, poor drug adherence, previous TB treatment failure, alcohol consumption, and being farmer were found as major factors for the development MDR-TB in Ethiopia [12, 13].

Notably, there is considerable empirical support for the claim that the cost of curing MDR-TB can be staggering thousands of times as expensive as that of regular treatment [14]. Importantly, according to WHO recommendation for programmatic management of drug-resistant tuberculosis, there is an urgent need of expansion of rapid testing and detection of MDR-TB cases, a target of one laboratory with the capacity to undertake drug susceptibility test for every five million people in the population. However, more than half of the 36 countries (including Ethiopia) with high TB and MDR-TB burden failed to meet this goal [14].

Hence, current knowledge on the prevalence of MDR is significantly critical to provide useful information for the implementation of standard chemotherapy regimens designed and recommended by WHO for TB patients who have or have not been treated previously [6, 7, 15].

Remarkably, there were limited data on the prevalence and associated factors with the occurrence of MDR-TB in the literature. Addressing these issues requires time and commitment on the part of researchers. Considering these aims, the present study was conducted to determine the prevalence and factors associated with MDR-TB among presumptive MDR-TB cases in Tigray Region, Ethiopia.

## 2. Materials and Methods

### 2.1. Study Design, Period, and Eligibility Criteria

**Figure 1:**
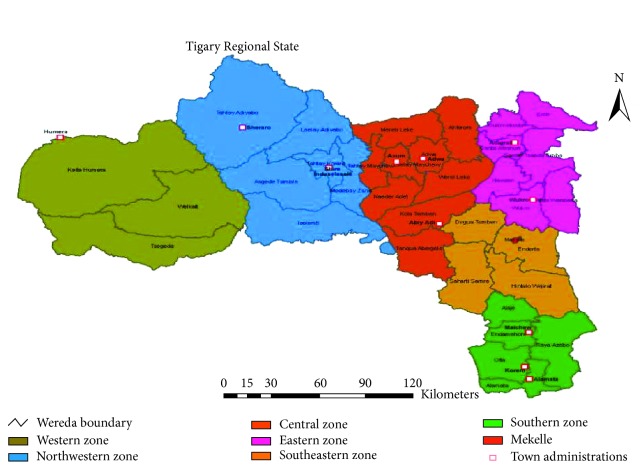
Samples collection site from all zones of Tigray Regional State.

A cross-sectional study was conducted in Tigray Regional State, Ethiopia, in 2015 to 2016. Tigray Regional State covers 54,572.6 square kilometers. The region is administratively divided into 7 zones, 52 weredas (34 rural and 18 urban), and 763 kebeles/tabiyas (Figure 1). The sputum samples were collected from all the 7 zones of the region. The collected specimens (sputum) were processed at Tigray regional laboratory. According to the 2007 census conducted by the Central Statistical Agency of Ethiopia, the region had an estimated total population of 4,314,456; about 80% of the population lived in rural areas [16]. Directly observed treatment, short-course (DOTS) TB treatment services were being provided in all the health facilities. The region had three MDR-TB treatment initiation centres and 52 treatment follow-up centres [17].

TB patients with the following history were included in the study: (a) household contacts of documented MDR-TB patients who developed active TB disease; (b) failures of new regimens with first-line anti-TB drugs; (c) failure of retreatment regimens and chronic TB cases; and (d) exposure to a known MDR-TB case, relapse, and loss to follow-up.

### 2.2. Sample Size and Sampling Technique

Considering 15.3% prevalence for MDR-TB from a previous study conducted in Amhara Regional State, Ethiopia [6] and 5% precision (*d* = 0.05), and 95% level of confidence (*z* = 1.96), the sample size was calculated using single population proportion formula: *n* *=* (1.96/0.05) 2 *∗* 0.153 (1–0.153) = 200. Consequently, a total of 200 consecutive presumptive MDR-TB patients living in Tigray Regional State were included in the study.

### 2.3. Data and Specimen Collection Methods

Data on age, sex, HIV status, TB treatment history, and other information were collected using a standardized data collection procedure. Sputum samples were collected by using 50 mL falcon tubes. The collected sputum sample was packed and transported to Tigray regional laboratory in accordance with the WHO recommendation for the transport of a biological substance, category B, UN-3373. The sputum samples were processed within 7 days of its collection.

## 3. Sample Processing

### 3.1. Ziehl–Neelsen Staining Method

Smears from culture media or direct from the decontaminated sample were prepared for detection of AFB using the Ziehl–Neelsen staining (ZNS) method. The staining was performed according to the standard protocol for sputum smear. Briefly, the smeared slides were heated to fix. Subsequently, filtered carbol fuchsin was added to cover the entire slide. Using flame, the slides were slowly heated until steam comes out and left for 5 minutes. After this procedure, the slides were rinsed with water. Then, by using 3% acid alcohol, the smears were decolourized. After rinsing the slides thoroughly with water again, the decolourized smears were counterstained with methylene blue for 2 minutes. Finally, the smears were rinsed with water, air-dried, and examined for AFB under a 100x oil immersion lens. The slide with bacilli which appeared red on a blue background was reported as positive for AFB. In both methods, negative and positive control smears were stained concurrently to assess the quality of reagents and staining procedures [18, 19].

### 3.2. Culture for MTB on Lowenstein–Jensen (LJ) Media

The collected samples were processed by using 2% N-acetyl-L-cysteine-NaOH (NALC-NaOH) decontamination method (final NaOH concentration, 1%). After decontamination, the concentrated sediment was suspended in 1.0 ml sterile phosphate buffer (pH 6.8) for inoculation into LJ medium. Subsequently, 0.1–0.2 mL was added to the LJ medium for culture and identification. The conventional culture media were examined for growth twice a week for the first four weeks starting on day 3 to 5 postinoculation, and thereafter, once a week until the eighth week. Moreover, all specimens showing growth in the culture were confirmed by smear microscopy. The results of the microscopy were recorded and reported immediately as “culture positive for *Mycobacteri*a pending identification.” Subsequently, all cultures reported positive for *Mycobacteria* were tested for detection of MPT64 antigen using the SD Bioline immunochromatography test kit [20]. On egg-based media, they produce characteristic nonpigmented colonies, with a generally rough and dry appearance, simulating breadcrumbs.

### 3.3. Molecular Techniques Used for *Mycobacterium* Drug Resistance Detection

DNA extraction was carried out using chemical methods. Briefly, a loop full colony was mixed with 300 ml DNA/RNA free molecular grade water or 500 *μ*L sediment was centrifuged (Thermo Fisher Scientific, Heraeus Megafuge 16R) at 13,000 g for 15 min. Subsequently, the supernatant was discarded, and each pellet was suspended in 100 *μ*L of lysis buffer (A-LYS) and then incubated for 5 min at 90°C for further killing and lyses. Finally, 100 mL of neutralization buffer (B-NB) was added and centrifuged at 10,000 g for 5 min. Then, the supernatant was aspirated into sterile 1.5 mL of Eppendorf tube. Resistance to RMP and INH was done using a line probe assay (LPA). Master mix preparation, amplification, and hybridization were performed as recommended by the manufacturer [21].

### 3.4. Quality Assurance and Analysis

All laboratory analysis was carried out following standard operating procedures. Both the solid culture and LPA laboratory were validated by the National TB Reference Laboratory, and trained laboratory technologists did the procedure. Reference strains of *M. tuberculosis* H37Rv were used as quality control organisms throughout the LPA test. Moreover, both start and end controls were used during each batch of specimen processing and DNA extraction. Additionally, Lot Quality Assurance System was used for verification of new LPA reagents.

Data were entered, cleaned, and analyzed using the SPSS version 20 (SPSS Inc., Chicago, IL, USA). Variables were descriptively expressed by frequency and percentage. Comparisons between MDR-TB and/or RMP mono-resistance (MR) and non-MDR were done using the odds ratio with a 95% confidence interval (CI). Bivariate and multivariable logistic regression was used to identify factors statistically associated with the prevalence of MDR-TB. A *P* value of less than 0.05 was considered as statistically significant.

### 3.5. Ethical Considerations

The study protocol was evaluated and approved by the Research Ethics Review Committee of the College of Health Sciences, Mekelle University. Besides, a letter of permission was obtained from the Tigray Regional Health Bureau. Informed consent was obtained from all the study participants. Confidentiality was assured by making the data anonymous. Laboratory results that have a direct effect on the health of the participant were communicated with the respective patient and health professionals for definitive management.

## 4. Results

### 4.1. Sociodemographic Characteristics

A total of 200 presumptive MDR-TB cases were included in the study. Majority of the participants were males (60.5%). The age of the participants ranged from 9 to 75 years, with a mean age of 38.24 (38.24 ± 15.49 SD) years. More than one-fourth (27.5%) were illiterates, 110 (55%) were rural inhabitants, and 58 (29%) were farmers (Table 1).

### 4.2. Distribution of Drug-Resistant TB

**Figure 2:**
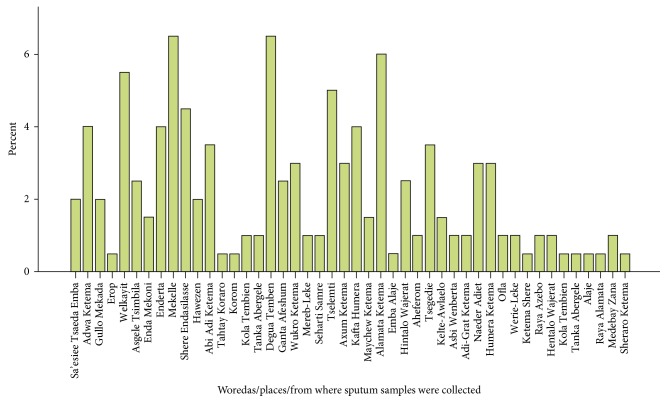
Distribution of sample collection in all zones of Tigray Regional State.

Among the 200 analyzed sputum specimens, 53 (26.5%) were smeared positive. Sputum culture result revealed that 74/200 (37.0%) were culture positive. Furthermore, the prevalence of MDR-TB was 37/200 (18.5%). However, considering RMP mono-resistance (RMP-MR) as a proxy indicator for MDR-TB, the prevalence of MDR-TB/RMP resistance was 7 (3.5%). The proportions of MDR-TB cases in each zone in a descending order were as follows: Western (31%), Southern (22.6%), Eastern (20%), Northwest (19.4%), Central (13.9%), Southeast (13.3%), and Mekelle Zone (0%) (Table 2 and Figure 2).

### 4.3. Level of Drug-Resistant TB

The prevalence of MDR-TB among culture-positive cases was 37/74 (50%). The prevalence of RMP and INH resistance was 44 (59.5%) and 42 (56.8%), respectively. Of these culture-positive cases, 25 (33.8%) were susceptible to both RMP and INH (Table 3).

### 4.4. Characteristics of the MDR-TB Cases

Impressively, the majority of the confirmed MDR-TB cases (23%) were in the age-group of 15–24 years, followed by 35–44 (20.5%) years. As it is shown in Table 4, 31 out of the 37 MDR-TB cases (83.8%) were treated at least once with first-line anti-TB drugs in their lifetime, while 30/37 (81%) of the MDR-TB cases were either case of relapse or had a failure of treatment. In terms of the occupation of the study participants, students, farmers, and housewives accounted for the greater part (67.5%) of the MDR-TB cases. All the MDR-TB cases were HIV negative.

### 4.5. Factors Associated with the MDR-TB Occurrence

After adjusting for potential confounding factors, age was statistically associated with MDR-TB (AOR: 1.06, 95% CI = 1.00–1.11). However, the occurrence of MDR-TB was not associated with sex, place of residence, educational status, not having homes without window, family size, income, alcohol intake, cigarette smoking, imprisonment, and TB treatment (Table 5).

## 5. Discussion

The growing prevalence of MDR-TB strains is a major public health problem globally and in Ethiopia; some studies reported that the prevalence of MDR-TB was almost nil or 1% around 15 years ago [22, 23]. Currently, increasing prevalence of MDR-TB was reported from different parts of the country [6, 15].

The prevalence of MDR-TB in this research was found to be 18.5%. This prevalence is higher than that of studies conducted in Amhara Regional State, Ethiopia (15.3%), Northwest Ethiopia (5.7%), and Iran (12.2%) [6, 15, 24]. On the other hand, the current prevalence is lower than the prevalence in Oromia, Ethiopia (20%) [7]. Though the prevalence of MDR-TB in Oromia Region in Ethiopia appears to be higher, the MDR-TB proportion in culture-positive isolates was only 33%, which is lower than that of the present study which was 50%. The possible explanations for these differences could be due to differences in study designs, target population, specimen quality, and processing.

The prevalence of MDR-TB among new and previously treated cases was found to be 1.5% and 17%, respectively. Conversely, much higher prevalence (16.9%) among newly suspected cases was reported by the study conducted in Amhara, Ethiopia [6]. Importantly, many pieces of research showed that MDR-TB was frequently identified in patients with a history of TB treatment, which is also evidenced in this study though not statistically associated [15, 25–29].

Furthermore, this study assessed factors associated with MDR-TB; age was significantly associated with MDR-TB (AOR: 1.06, 95% CI = 1.00–1.11). On the contrary, several studies showed the absence of statistically significant difference in the proportion of any resistance by age [15, 24]. Furthermore, the majority of the MDR-TB cases 86.5% (32/37) were in the age-group of 15 to 44 years, which is within the productive age-group. The productive age-group by MDR-TB in Ethiopia and other parts of the world has been reported [7, 13, 30, 31]. Even though this study revealed that only the age of the patient was “marginally” statistically associated with MDR-TB, several studies had reported that factors including the history of treatment, treatment category, HIV/AIDS, overcrowding, smoking, opportunistic infection, and lack of compliance with DOTS program were the potential risk factors for acquisition of MDR-TB infection [15, 32–34].

Considerably, the emergence of new cases with MDR-TB has frequently been related to close contact with known cases, facilitated by overcrowding [24]. Likewise, the present study showed that 48.6% of new MDR-TB cases were farmers and students though not statistically significant. Moreover, 35% of the MDR-TB cases had treatment failure. Unlike the present study, previous TB treatment was significantly associated with the prevalence of MDR-TB in Georgia [32].

## 6. Conclusion and Recommendations

The overall prevalence of MDR-TB was found to be high about 18.5% among presumptive MDR-TB cases. Age was marginally statistically associated with MDR-TB. This is a warning to TB control programme in Tigray Regional State, Ethiopia, so that preventive measures should be taken to prevent the transmission of MDR-TB in the community. Further operational studies should be conducted in the region to study the contribution of different risk factors including HIV coinfection for development of MDR-TB.

## Figures and Tables

**Table 1:** Sociodemographic characteristics of presumptive MDR-TB cases in Tigray Regional State, Ethiopia.

Variable		Number	Percentage
Age (years)		38.24 ± 15.49^#^	[9–75]^##^

Sex (male)		121	60.5

Address	Urban	90	45
	Rural	110	55

Educational status	Unable to read and write	55	27.5
	Read and write only	30	15
	Elementary (1–6)	30	15
	Secondary (7–12)	68	34
	Diploma	13	6.5
	Degree	4	2

Occupation	Farmer	58	29
	Housewife	50	25
	Student	32	16
	Merchant	29	14.5
	Teacher	11	5.5
	Soldier	5	2.5
	Others^*∗*^	15	7.5

Family size	Less than or equal to 4	95	47.5
	Above 4	105	52.5

^*∗*^Others: finance, secretary, human resource, priest, office assistant, daily laborer, and dependent on others; ^#^mean ± standard deviation; ^##^range.

**Table 2:** The distribution of mono- and multidrug-resistant TB cases in Tigray Regional State, Ethiopia.

Zone	Presumptive MDR-TB cases	Sputum smear positive for AFB	TB culture-positive cases	Isoniazid-only resistant cases	Rifampicin mono-resistant cases	MDR-TB cases
	No. (%)	No. (%)	No. (%)	No. (%)	No. (%)	No. (%)
Mekelle	13 (6.5)	2 (15.4)	4 (30.8)	0 (0.0)	0 (0.0)	0 (0.0)
Northwest	31 (15.5)	10 (32.3)	11 (35.5)	0 (0.0)	2 (6.5)	6 (19.4)
Southern	31 (15.5)	13 (41.9)	16 (51.6)	2 (6.5)	1 (3.2)	7 (22.6)
Southeast	30 (15.0)	7 (23.3)	12 (40.0)	2 (6.7)	3 (10.0)	4 (13.3)
Central	36 (18.0)	5 (13.9)	8 (22.2)	0 (0.0)	0 (0.0)	5 (13.9)
Western	29 (14.5)	8 (27.6)	12 (41.4)	0 (0.0)	0 (0.0)	9 (31.0)
Eastern	30 (15.0)	8 (26.7)	11 (36.7)	1 (3.3)	1 (3.3)	6 (20.0)
Total	**200 (100.0)**	**53 (26.5)**	**74 (37.0)**	**5 (2.5)**	**7 (3.5)**	**37 (18.5)**

**Table 3:** Resistance pattern of *Mycobacterium tuberculosis* to isoniazid and/or rifampicin among TB culture-positive versus presumptive MDR-TB cases in Tigray Regional State, Ethiopia.

Anti-TB drugs	TB culture-positive cases, *N* = 74		Presumptive MDR-TB cases, *N* = 200	
	Number	Percentage	Number	Percentage
Resistant to rifampicin^*∗*^ (RMP)	44	59.5	44	22.0
Resistant to isoniazid^*∗∗*^ (INH)	42	56.8	42	21.0
Resistant to RMP and INH	37	50.0	37	18.5
Sensitive to RMP and INH	25	33.8	25	12.5
RMP mono-resistance	7	9.5	7	3.5
INH only resistance	5	6.8	5	2.5

^*∗*^Rifampicin only and MDR- TB cases; ^*∗∗*^isoniazid only and MDR-TB cases; “*N*” is the number of cases in the respective column.

**Table 4:** Characteristics of the MDR-TB cases (*N* = 37) in Tigray Regional State, Ethiopia.

Variable		Number	Percentage
Age (years)		29.9 ± 11.9^§^	[9–67]^§§^

Sex (male)	Male	22	59.5

Address	Urban	16	43.2
	Rural	21	56.8

Educational status	Unable to read and write	8	21.6
	Read and write only	4	10.8
	Elementary (1–6)	12	32.4
	Secondary (7–12)	11	29.7
	Diploma	2	5.4

Occupation	Farmer	9	24.3
	House wife	7	18.9
	Student	9	24.3
	Merchant	5	13.5
	Daily laborer	2	5.4
	No work	2	5.4
	Others^*∗*^	3	8.1

Ethnicity	Tigraway/ti	36	97.3
	Amhara	1	2.7

Family size	Less than or equal to 4	10	27
	Above 4	27	73

Monthly expenditure (birr)		848.4 ± 578.0^§^	[150–2400]^§§^

TB treatment history	New	6	16.2
	Relapse	17	45.9
	After defaulter	1	2.7
	After failure of treatment	13	35.1

First-line anti-TB treatment	None	3	8.1
	Currently on first-line anti-TB	3	8.1
	Once	3	8.1
	Two times	15	40.5
	Three times	10	27
	Four times	3	8.1

^§^Mean ± standard deviation; ^§§^range; ^*∗*^others refers to a teacher, human resource, and finance personnel.

**Table 5:** Factors associated with MDR-TB in Tigray Regional State, Ethiopia.

Variable		MDR-TB cases, *N* = 37		Crude OR (95% CI)	*P* value	Adjusted OR (95% CI)	*P* value
		No.	%				
Sex	Female	15	40.5	1	0.632	1	0.876
	Male	22	59.5	1.26 (0.49–3.23)		1.13 (0.249–5.09)	

Age (years)		29.9 + 11.9^§^	[9–67]^§§^	1.06 (1.02–1.10)	0.005	1.06 (1.00–1.11)	0.048

Address	Rural	21	56.8	1	0.814	1	0.392
	Urban	16	43.2	0.90 (0.36–2.25)		0.55 (0.14–2.17)	

Educational status	Unable to read and write	8	21.6	1		1	
	Read and write only	4	10.8	1.23 (0.28–5.45)	0.785	0.81 (0.12–5.33)	0.827
	Elementary (1–6)	12	32.4	0.26 (0.07–1.00)	0.051	0.23 (0.03–1.54)	0.129
	Secondary (7–12)	11	29.7	0.39 (0.11–1.43)	0.156	0.55 (0.11–2.72)	0.463
	Diploma	2	5.4	1.23 (0.18–8.33)	0.831	0.33 (0.02–7.14)	0.483

Window	No	11	29.7	1	0.601	1	0.567
	Yes	26	70.3	1.32 (0.47–3.69)		1.54 (0.35–6.72)	

Family size	Less than or equal to 4	10	27	1	0.611	1	0.547
	Above 4	27	73	0.77 (0.28–2.10)		1.59 (0.35–7.21)	

Monthly expenditure (birr)		848.4 + 578.0^§^	[150–2400]^§§^	1.00 (1.000–1.001)	0.314	1.00 (0.99–1.00)	0.722

Alcohol consumption	No	20	54.1	1	0.353	1	0.525
	Yes	17	45.9	1.54 (0.62–3.86)		0.61 (0.13–2.83)	

Cigarette smoking	No	34	91.9	1	0.070	1	0.118
	Yes	3	8.1	3.64 (0.90–14.76)		6.94 (0.61–78.58)	

Imprisonment	No	34	91.9	1	1.000	1	0.867
	Yes	3	8.1	1.00 (0.19–5.31)		0.83 (0.93–7.43)	

TB treatment category	New	6	16.2	1		1	
	Relapse	17	45.9	0.82 (0.22–3.13)	0.776	5.66 (0.37–87.72)	0.215
	After defaulter	1	2.7	2.00 (0.14–28.42)	0.609	2.30 (0.03–161.00)	0.701
	After failure of treatment	13	35.1	1.15 (0.30–4.47)	0.836	8.38 (0.47–150.08)	0.149

TB treatment	Never treated	3	8.1	1		1	0.097
	Ever treated	34	91.9	3.18 (0.32–32.04)	0.327	16.97 (0.60–480.02)	

^§§^Mean ± standard deviation; *P* values generated using either chi-square test or Fisher's exact test.

## Data Availability

The data used to support the findings of this study are available from the first author and corresponding author upon request.

## References

[1] WHO (2012). Global tuberculosis report 2012.

[2] Van Soolingen D. (2001). Molecular epidemiology of tuberculosis and other mycobacterial infections: main methodologies and achievements. *Journal of Internal Medicine*.

[3] Boehme C. C., Nabeta P., Hillemann D. (2010). Rapid molecular detection of tuberculosis and rifampin resistance. *New England Journal of Medicine*.

[4] WHO (2015). Global tuberculosis report 2015.

[5] WHO (2014). Global tuberculosis report 2014.

[6] Nigus D. M., Lingerew W. M., Beyene B. A., Tamiru A. A., Lemma M. T., Melaku M. Y. (2014). Prevalence of multi drug resistant tuberculosis among presumptive multi drug resistant tuberculosis cases in Amhara National Regional State, Ethiopia. *Mycobacterial Diseases*.

[7] Abate D., Taye B., Abseno M., Biadgilign S. (2012). Epidemiology of anti-tuberculosis drug resistance patterns and trends in tuberculosis referral hospital in Addis Ababa, Ethiopia. *BMC Research Notes*.

[8] Saravanan M., Niguse S., Abdulkader M. (2018). Review on emergence of drug-resistant tuberculosis (MDR & XDR-TB) and its molecular diagnosis in Ethiopia. *Microbial Pathogenesis*.

[9] Calver A. D., Falmer A. A., Murray M. (2010). Emergence of increased resistance and extensively drug-resistant tuberculosis despite treatment adherence, South Africa. *Emerging Infectious Diseases*.

[10] Joseph P., Rao V. B., Mohan N. S. (2012). Outcome of standardized treatment for patients with MDR-TB from Tamil Nadu, India. *Indian Journal of Medical Research*.

[11] WHO (2016). Global tuberculosis report 2016.

[12] Mulisa G., Workneh T., Hordofa N., Suaudi M., Abebe G., Jarso G. (2015). Multidrug-resistant *Mycobacterium tuberculosis* and associated risk factors in Oromia Region of Ethiopia. *International Journal of Infectious Diseases*.

[13] Mulu W., Mekkonnen D., Yimer M., Admassu A., Abera B. (2015). Risk factors for multidrug resistant tuberculosis patients in Amhara National Regional State. *African Health Sciences*.

[14] WHO (2010). *Global Tuberculosis Control: Surveillance, Planning, Financing*.

[15] Mekonnen F., Tessema B., Moges F., Gelaw A., Eshetie S., Kumera G. (2015). Multidrug resistant tuberculosis: prevalence and risk factors in districts of Metema and West Armachiho, Northwest Ethiopia. *BMC Infectious Diseases*.

[16] Central Statists Agency [Ethiopia] (2007). Population and housing census report-country. http://www.csa.gov.et/newcsaweb/images/documents/surveys/PopulationandHousingcensus/ETH-pop-2007/survey_datareportstigray.pdf.

[17] Tigray Regional Health Bureau (2007). *TRHB Annual Profile 2007 EFY*.

[18] Cheesbrough M. (2006). *District Laboratory Practice in Tropical Countries; Part 2*.

[19] Hernandez-Garduno E., Cook V., Kinimoto D., Elwood R. K., Black W. A. (2004). Transmission of tuberculosis from smear negative patients: a molecular epidemiology study. *Thorax*.

[20] Kumar V. G. S., Urs T. A., Ranganath R. R. (2011). MPT 64 antigen detection for rapid confirmation of *M. tuberculosis* isolates. *BMC Research Notes*.

[21] Anupurba S., Tripathi R., Sinha P., Kumari R., Chaubey P., Pandey A. (2016). Detection of rifampicin resistance in tuberculosis by molecular methods: a report from Eastern Uttar Pradesh, India. *Indian Journal of Medical Microbiology*.

[22] Bruchfeld J., Aderaye G., Palme I. B. (2002). Molecular epidemiology and drug resistance of *Mycobacterium tuberculosis* isolates from Ethiopian pulmonary tuberculosis patients with and without human immunodeficiency virus infection. *Journal of Clinical Microbiology*.

[23] Demissie M., Lemma E., Gebeyehu M., Lindtjorn B. (2001). Sensitivity to anti-tuberculosis drugs in HIV-positive and -negative patients in Addis Ababa. *Scandinavian Journal of Infectious Diseases*.

[24] Metanat M., Sharifi-Mood B., Shahreki S., Dawoudi S. H. (2012). Prevalence of multidrug-resistant and extensively drug-resistant tuberculosis in patients with pulmonary tuberculosis in Zahedan, Southeastern Iran. *Iranian Red Crescent Medical Journal*.

[25] Kebede A. H., Alebachew Z., Tsegaye F. (2014). The first population-based national tuberculosis prevalence survey in Ethiopia, 2010-2011. *The International Journal of Tuberculosis and Lung Disease*.

[26] Tessema B., Beer J., Emmrich F., Sack U., Rodloff A. C. (2012). First- and second-line anti-tuberculosis drug resistance in Northwest Ethiopia. *The International Journal of Tuberculosis and Lung Disease*.

[27] WHO (2015). *Towards Universal Access to Diagnosis and Treatment of Multidrug-Resistant and Extensively Drug-Resistant Tuberculosis by 2015*.

[28] Adane K., Ameni G., Bekele S., Abebe M., Aseffa A. (2015). Prevalence and drug resistance profile of *Mycobacterium tuberculosis* isolated from pulmonary tuberculosis patients attending two public hospitals in East Gojjam zone, Northwest Ethiopia. *BMC Public Health*.

[29] Maru M., Mariam S. H., Airgecho T., Gadissa E., Aseffa A. (2015). Prevalence of tuberculosis, drug susceptibility testing, and genotyping of mycobacterial isolates from pulmonary tuberculosis patients in Dessie, Ethiopia. *Tuberculosis Research and Treatment*.

[30] Rifat M., Milton A. H., Hall J. (2014). Development of multidrug resistant tuberculosis in Bangladesh: a case-control study on risk factors. *PLoS ONE*.

[31] Biadglegne F., Sack U., Rodlof A. C. (2014). Multidrug-resistant tuberculosis in Ethiopia: efforts to expand diagnostic services, treatment and care. *Antimicrobial Resistance and Infection Control*.

[32] Vashakidze L., Salakaia A., Shubladze N. (2009). Prevalence and risk factors for drug resistance among hospitalized tuberculosis patients in Georgia. *International Journal of Tuberculosis and Lung Disease*.

[33] Abdella K., Abdissa K., Kebede W., Abebe G. (2015). Drug resistance patterns of *Mycobacterium tuberculosis* complex and associated factors among retreatment cases around Jimma, Southwest Ethiopia. *BMC Public Health*.

[34] Berhan A., Berhan Y., Yizengaw D. (2013). A meta-analysis of drug resistant tuberculosis in Sub-Saharan Africa: how strongly associated with previous treatment and HIV co-infection?. *Ethiopian Journal of Health Sciences*.

